# Temporally Delayed Deployment of Photo‐Responsive Liquid Crystal Polymer Networks Toward Neural Interfaces

**DOI:** 10.1002/adhm.202505422

**Published:** 2026-03-14

**Authors:** Yeh‐Chia Tseng, Eleanor Nguyen Jeakle, Mahjabeen Javed, Rajasree Gopala Krishnan, Thomas J. Smith, Yoo Jin Lee, Sahana Dhananjayan, Sasha M. George, Manivannan Sivaperuman Kalairaj, Ali Hasan Zuaiter, Majed O. Althumayri, Hatice Ceylan Koydemir, Joseph J. Pancrazio, Taylor H. Ware

**Affiliations:** ^1^ Department of Biomedical Engineering Texas A&M University College Station Texas USA; ^2^ Department of Bioengineering The University of Texas at Dallas Richardson Texas USA; ^3^ School of Behavioral and Brain Sciences The University of Texas at Dallas Richardson Texas USA; ^4^ Department of Material Sciences and Engineering Texas A&M University College Station Texas USA; ^5^ Center for Remote Health Technologies and Systems Texas A&M Engineering Experiment Station College Station Texas USA

**Keywords:** actuators, azobenzene, liquid crystal networks, medical devices, photo‐responsive, shape‐changing

## Abstract

Deployable medical devices are designed to be compact during insertion and expand after surgical placement. Devices such as neural interfaces can leverage deployment to minimize the size of the foreign body cascade near the electrode, potentially improving chronic recording and stimulation performance. To trigger deployment, a stimuli responsive material can be used. However, external stimuli are difficult to supply within tissues. Intrinsic changes upon implantation, such as water uptake, are difficult to control and may lead to device failure. Here, we describe a strategy to deploy small‐scale structures into soft tissues after insertion without the need for any stimulus. Photoresponsive liquid crystal networks (LCNs) are crosslinked after self‐assembly of monomers and adopt a programmed 3D form at room temperature (RT). The *trans‐cis* isomerization of azobenzene enables the 3D LCN films to be flattened by UV light before insertion and revert to 3D forms over 5 h at body temperature. A film programmed to adopt a cone shape with a diameter of 531 µm can actuate to 53 µm in height. Rigidity of the films enables penetration into and deployment within soft tissues. The described materials could potentially enable self‐deployable biomedical devices, including neural interfaces with sub‐mm features.

## Graphical abstract

Deployable medical devices typically need external stimuli to trigger deployment. However, external stimuli are difficult to supply within tissues. Here, we describe a strategy to deploy small‐scale structures into soft tissues after insertion without the need for any stimulus. We demonstrate deployment within a tissue phantom.

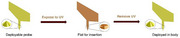

## 1 Introduction

Stimuli‐responsive materials have been used to make a wide range of deployable medical devices that are inserted in a compact form and then deploy into a 3D form. For example, superelasticity is leveraged to make shape memory alloy stents that deploy instantly after release from confinement within a catheter [1]. Shape memory polymer foams that expand on exposure to moisture or heat can also be deployed to serve as embolic agents [2, 3, 4]. A wide range of medical devices has been proposed using similar phenomena [5, 6, 7, 8, 9, 10, 11]. Several critical factors must be considered in the design of these materials. In addition to device‐specific considerations, the type of deployment that can be achieved and the trigger that causes deployment must be engineered. Frequently, the shape change that is achieved is programmed by mechanical deformation of the manufactured device into a temporary form, which we describe as packaging. Triggers that may induce deployment are, most frequently, release from an insertion device [12, 13, 14], a temperature change from ambient to physiological conditions [6, 8, 10, 15, 16], or exposure to physiological fluids [7, 17, 18]. However, these stimuli are difficult to control. Water uptake is detrimental to many bioelectronic devices [19, 20, 21]. Furthermore, small deployable devices (sub‐mm) are challenging to package mechanically. Materials are needed that can deploy after surgical placement without requiring an external stimulus or rely on water uptake.

Neural interfaces are devices that may be substantially improved by deployment. Frequently, intracortical electrodes fail to record extracellular potentials over chronic time periods [20, 22, 23]. There are both biotic and abiotic failure mechanisms. Upon implantation into cortical tissues, neural interfaces trigger a neuroinflammatory response resulting in glial encapsulation. This glial response region is generally 50–100 µm surrounding the implant site [12, 24, 25, 26]. Due to the initial surgical trauma and neuroinflammatory response, there is neuron loss at the implant site [26, 27, 28]. Cellular‐sized electronic threads have been shown to largely eliminate the glial scar response [29, 30]. However, these very flexible structures can be difficult to place in the tissue in controlled ways [31]. To overcome these challenges, several deployable neural probes have been reported [13, 32, 33, 34, 35]. One approach involves the use of large structures that can be inserted into the tissue, but these devices have relatively small electrodes that then deploy away from the initial insertion region. In principle, these small electrodes surpass the primary glial encapsulation range exhibited by the larger structures, with the goal of improving chronic recording performance. Egert et al. developed a silicon‐based deployable neural probe with springs. The springs were first retracted and held with dissolvable glue to enable insertion in a compact form. After implantation, the glue dissolved, and the electrode sites deployed from the shank [32]. Jiao et al. showed a deployable probe attached to a silicon shank with a magnesium sacrificial layer adhering the two structures. Once the sacrificial layer was dissolved, the deployable structure released [13]. Nonetheless, these approaches used insertion aids, which make the insertion effects occur in a larger dimension than the probe itself, possibly magnifying the initial tissue injury to the brain [20, 36, 37].

The development of self‐deployable neuronal electrodes may improve insertion‐related concerns. For example, Xie et al. developed macroporous probes with subcellular‐sized deployable structures. Using surface tension, these structures were pulled back to a mesh form to allow a smooth probe surface in air and then quickly frozen in liquid nitrogen to retain the shape and enhance rigidity for insertion. Once the probe was thawed in a moist environment, these structures would deploy [33]. Responsive polymers, such as shape memory polymers (SMP), can change shape and elastic modulus after insertion [26, 31, 38, 39]. The stimuli that trigger material changes are typically water uptake or heat. Sharp et al. created an SMP‐based self‐deploying neuronal electrode for slow insertion to minimize the glial scar. The probe was pre‐deformed into a crouched shape and slowly deployed when it transitioned from room to physiologic temperatures [35]. Wang et al. developed mechanically adaptive deployable neural probes using temperature‐responsive SMP. The probes were designed to be fishbone structures that could be inserted into a brain‐like phantom without using insertion aids. The probes remained folded during insertion at high stiffness and deployed to an unfolded state at physiologic temperature post‐implantation [31]. These examples provide approaches to create self‐deployable neural probes without using insertion aids. However, using moisture and heat as stimuli can lead to premature deployment before the probes are fully implanted. Furthermore, moisture uptake in the polymer network degrades the performance of the material as a dielectric, which is a key mode of abiotic device failure [40, 41].

Liquid crystal polymer networks (LCNs) show potential to be deployable materials for medical devices [42, 43, 44, 45, 46]. Liquid crystal polymers exhibit low water uptake, which has led to interest in medical electronics packaging [45, 47, 48]. Aligned LCNs, which are densely crosslinked liquid crystal polymers and the related class of materials known as liquid crystal elastomers (LCEs), can undergo reversible shape changes in response to stimuli such as heat [49, 50] and light [51, 52, 53, 54]. Surface alignment can be used to program complex molecular orientation for LCNs [49]. For this class of materials, the nematic director, the average orientation of liquid crystal molecules, can be spatially controlled using the surfaces of a mold. The monomer then adopts this orientation, which is ultimately trapped by crosslinking. Upon heating, there is a reversible, local contraction along the nematic director. In materials with a patterned nematic director, the material can undergo programmed deformations such as bending [55, 56], twisting [57, 58], stretching into cones [49, 59, 60], and other forms [61]. When utilizing these procedures, aligned LCNs can serve as programmable substrates for planar microelectronics fabrication methods. In this scenario, the electronics are processed when the LCNs are in a flat form on a carrier substrate. After processing, the shape change of the LCNs can cause the electronic device to adopt a 3D shape [57]. Crosslinking the LCNs at high temperatures allows for the programmed shape change to occur by cooling to ambient or physiologic temperatures. However, the approach does not allow for controlled deployment. Alternatively, azobenzene‐containing monomers can also be incorporated into the LCNs to induce shape change [62]. In those materials, the *trans‐cis* isomerization of azobenzene is triggered upon UV irradiation, which leads to the linear *trans* azobenzene transformation to the bent *cis* azobenzene. The reduction in order caused by the *cis* isomer of azobenzene in the LCNs causes shape deformation [63, 64].

In this study, LCNs with controlled alignment are crosslinked at high temperatures and then cooled to show a 3D form at room and body temperature. We utilize the photoresponsiveness of azobenzene‐containing LCNs to package devices with a planar form and rely on the spontaneous *cis‐trans* isomerization of azobenzene to cause 3D deployment, without the need for a stimulus. The effect of environmental moisture and temperature on deployment is characterized. We then pattern sub‐mm features into LCNs and characterize their insertion and deployment in tissue‐like environments.

## 2 Results and Discussion

### 2.1 Synthesis and Characterization of Liquid Crystal Networks

**FIGURE 1:**
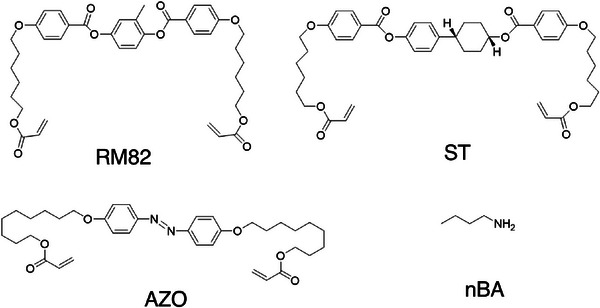
Chemical structure of the monomers used in this study. RM82: 1,4‐Bis[4‐(6‐acryloyloxyhexyloxy)benzoyloxy]‐2‐methylbenzene, ST: 6‐{4‐[4‐(Acryloyloxy‐hexyloxy)cyclohexyl]phenoxyhexyl acrylate, AZO: 4,4'‐Bis(9‐(acryloyloxy)nonyloxy)azobenzene, nBA: n‐butylamine.

The molecular structures of the monomers used for synthesis are shown in Figure 1. The synthesis strategy for the fabrication of LCNs using acrylate‐amine chemistry has been described in previous studies [49]. These monomers first undergo oligomerization by Michael addition, yielding short, acrylate‐terminated oligomers. These oligomers are then crosslinked into polymer networks using free radical polymerization. We use a relatively high acrylate to amine ratio of 2.3 such that the final films have high crosslink density, which in turn yields networks with glass transition temperatures above 37°C. This chemistry is also amenable to surface alignment methods, which allow the nematic director, and therefore the shape change of the LCNs, to be programmed [60]. Azobenzene incorporation allows for the material to be rendered photoresponsive.

**FIGURE 2:**
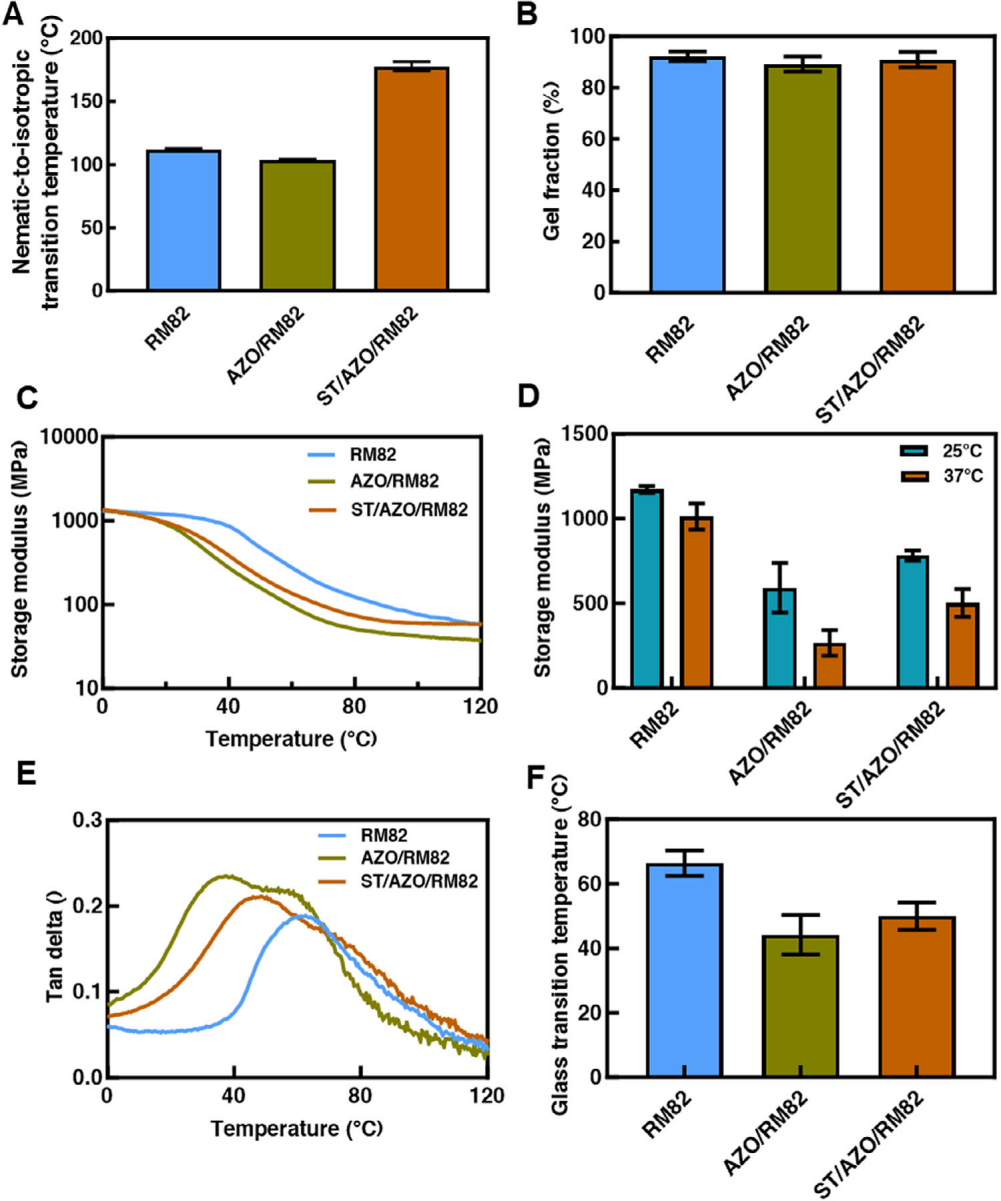
(A) Nematic‐to‐isotropic transition temperature after oligomerization (B) Gel fraction for RM82, AZO/RM82, and ST/AZO/RM82 LCNs (C) Storage modulus as a function of temperature (D) Storage modulus summary at 25°C and 37°C (E) Tan delta as a function of temperature, and (F) Glass transition temperature for RM82, AZO/RM82, and ST/AZO/RM82 LCNs. Data shown are representative of three replicates. Data shown are the mean with error bars representing standard deviation (*n* = 3).

The nematic‐to‐isotropic temperature (*T*_NI_) of the monomer mixture controls the temperature range of processing and the equilibrium shape of the material at 37°C. The shape in LCNs crosslinked in the oriented, nematic state is a property that varies continuously with temperature [57, 60, 65]. By crosslinking the material at a relatively high temperature and then cooling the material to room or body temperature leads to a shape change that can be used to make a 3D structure. The strain in the material achieved on cooling can be maximized if the temperature difference between the crosslinking temperature and the use temperature is maximized [57]. Here, we fabricated LCNs using three different compositions where the acrylate monomers were varied. The *T*_NI_ of the RM82 composition after oligomerization was 112 ± 1°C (RM82). The *T*_NI_ slightly decreased to 104 ± 1°C as we incorporated the photo‐responsive monomer AZO in the mixture (AZO/RM82). This drop in *T*_NI_ may be attributed to the molecular structure of azobenzene, which slightly disrupts the order of the material. Monomer ST can effectively increase the *T*_NI_ of the monomer mixture [66]. The addition of monomer ST dramatically increased the mixture T_NI_ to 178 ± 4°C (ST/AZO/RM82) (mean ± STD, *n* = 3) (Figure 2A). After photocrosslinking the oligomers, the samples all had over 85% gel fraction (Figure 2B). The storage moduli of RM82, AZO/RM82, and ST/AZO/RM82 samples are 1013 ± 76, 267 ± 76, and 503 ± 82 MPa at 37°C (mean ± STD, n = 3), respectively (Figure 2C,D). The *T*_g_ of RM82, AZO/RM82, and ST/AZO/RM82 samples are 66 ± 4°C, 44 ± 6°C, and 50 ± 4°C, respectively (mean ± STD, *n* = 3) (Figure 2E,F), indicating that they were in the glassy or viscoelastic state at room and body temperature. Elastic modulus in this range may allow for penetration of some soft tissues without buckling, depending on the size of the device.

### 2.2 Photoresponse of LCN Benders

**FIGURE 3:**
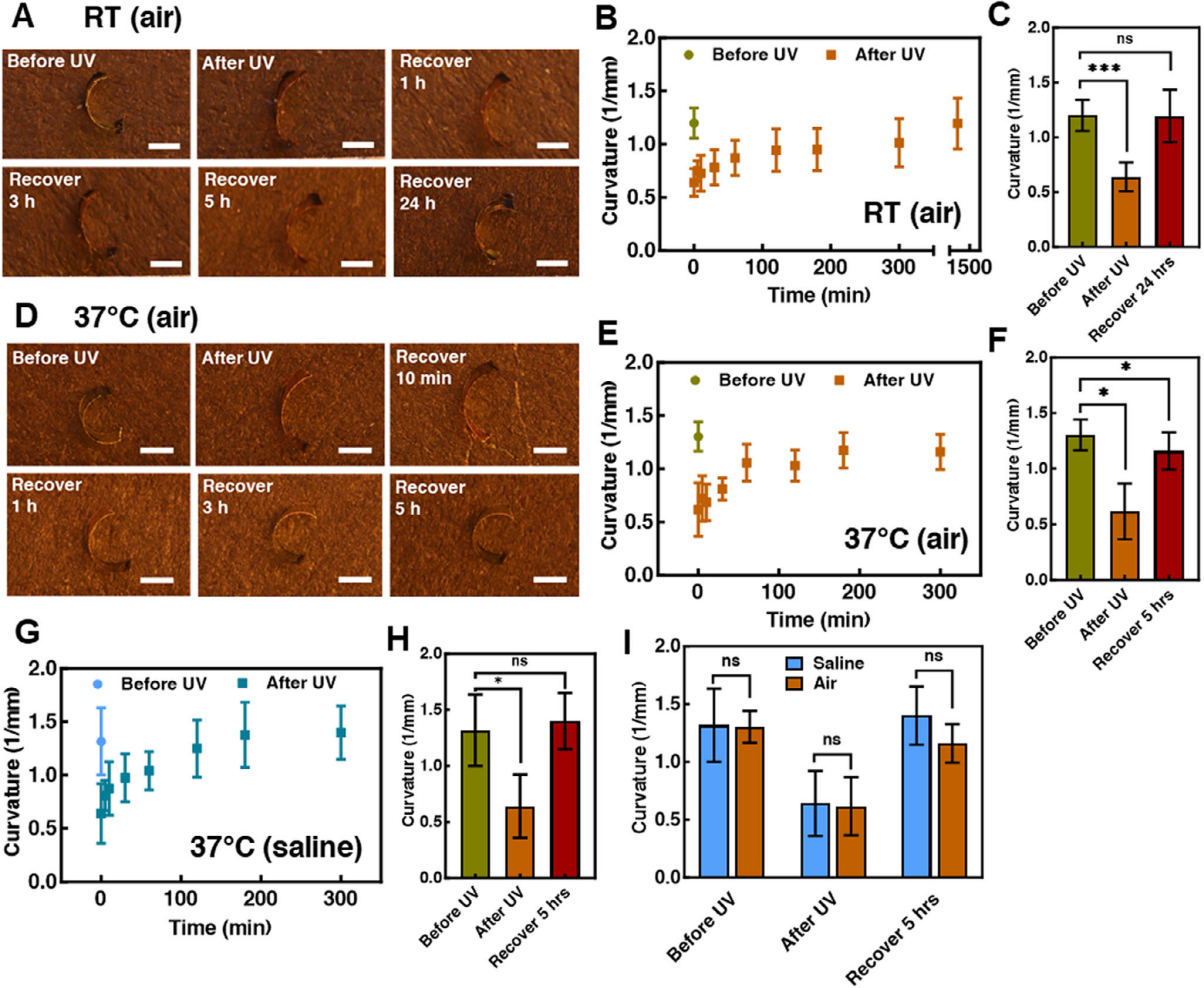
(A) Time‐dependent recovery of ST/AZO/RM82 twisted nematic samples in air at RT. (Scale bar: 1 mm) (B) Curvature change over time of ST/AZO/RM82 twisted nematic samples in the air at RT (C) Curvature change before UV, right after UV, and after recovering for 24 h at RT. One‐way ANOVA was performed with Dunnett's comparison. (D) Time‐dependent recovery of ST/AZO/RM82 twisted nematic samples in air at 37°C. Scale bar: 1 mm. (E) Curvature changes at 37°C in air over time. (F) Curvature change before UV, right after UV, and after recovering for 5 h at 37°C under air. One‐way ANOVA was performed with Dunnett's comparison. (G) Curvature changes at 37°C in saline over time. (H) Curvature change before UV, right after UV, and after recovering for 5 h at 37°C in saline. One‐way ANOVA was performed with Dunnett's comparison. (I) Statistics of curvature between air and saline environment at 37°C. Unpaired *t*‐tests were performed assuming the datasets follow a normal distribution between air and saline for each time point. All time points showed no significant difference between the curvature in air and saline environment (*p* > 0.05). ns: *p* > 0.05, ^*^ *p* < 0.05, ^**^ *p* < 0.01, and ^***^ *p* < 0.001. Data shown are representative of three replicates. Data shown are the mean with error bars representing standard deviation (*n* = 3).

Azobenzene‐containing LCNs undergo photoinduced shape change. By exposure to UV light, *trans* azobenzene converts to the *cis* form and spontaneously reverts over time in the dark [55, 56, 59, 67, 68, 69, 70, 71, 72, 73]. In this work, our goal is to use the spontaneous *cis‐trans* isomerization and the resulting shape change to deploy a medical device. Hence, it is important to evaluate the photo‐responsiveness upon UV exposure and recovery time after UV exposure for ST/AZO/RM82 LCNs. We first fabricated twisted‐nematic films of ST/AZO/RM82 and cut the materials into ribbons with the nematic director at the surfaces aligned along the long and short axes of the ribbons. All films are bent at RT as expected [12, 55, 56, 74] ST/AZO/RM82 LCN samples exposed to UV light morph from more to less bent forms and then slowly recover in the dark. We emphasize that both sides of the LCN films are exposed to UV light. Irradiation from one side induces bending, as the strong absorption of the azobenzene causes the photoisomerization of the LCN films to vary across the film thickness [75, 76, 77]. Samples were briefly moved to an environment without blue and blue‐green light to take images. The ST/AZO/RM82 LCN films fully recovered within 24 h at RT in air after terminating UV exposure (Figure 3A–C). Compared with the curvature before UV, the curvature after UV exposure showed a statistically significant decrease (*p* < 0.001). Curvature after recovering 24 h revealed no significant difference with the curvature before UV exposure (*p* > 0.05) (Figure 3C). The ST/AZO/RM82 LCNs nearly fully recovered within 5 h at 37°C in air (Figure 3D–F) or saline (Figure 3G,H) after terminating UV exposure. We highlight the fact that saline did not appear to affect the recovery process (*p* > 0.05) (Figure 3I). A water uptake study was performed to evaluate the water absorption of ST/AZO/RM82 LCNs. The LCNs swelled by 0.5% ± 0.8% in 7 days (mean ± STD, *n* = 3). This water uptake was not statistically significant (*p* > 0.05). After drying, the dry mass decreased by 0.1% ± 0.6% (mean ± STD, n = 3) as compared to the initial sample mass. This change in dry mass was also not significant (*p* > 0.05). These results suggest that the LCN films show potential to be used in physiological environments without experiencing significant water absorption or mass loss in water. The material does not rely on any temperature change, light exposure, or water uptake to drive recovery. As such, this spontaneous recovery process is promising for the deployment of small‐scale structures within living tissues where the application of stimuli is fraught with many challenges.

There are several practical considerations regarding using this recovery mechanism for a biomedical device. Recovery occurs spontaneously at room temperature, as shown in Figure 3A,B. This would necessitate irradiation of the device within minutes to hours prior to implantation. We focused on recovery driven by the photochemical effect. During irradiation, some of the energy absorbed by the film results in heating, which also causes shape change, disrupting the molecular order [75, 78, 79]. The choice of irradiation conditions substantially controls the photothermal effect. We note that in films with micrometer‐scale thickness, passive cooling is rapid. The measurements in this study were all collected after cooling to the temperature indicated. The strong absorption of azobenzene also provides a limitation on the thickness, in the range of 10's µm, of any device to use this deployment mechanism.

### 2.3 Photoresponse of LCNs with 3D Cones

**FIGURE 4:**
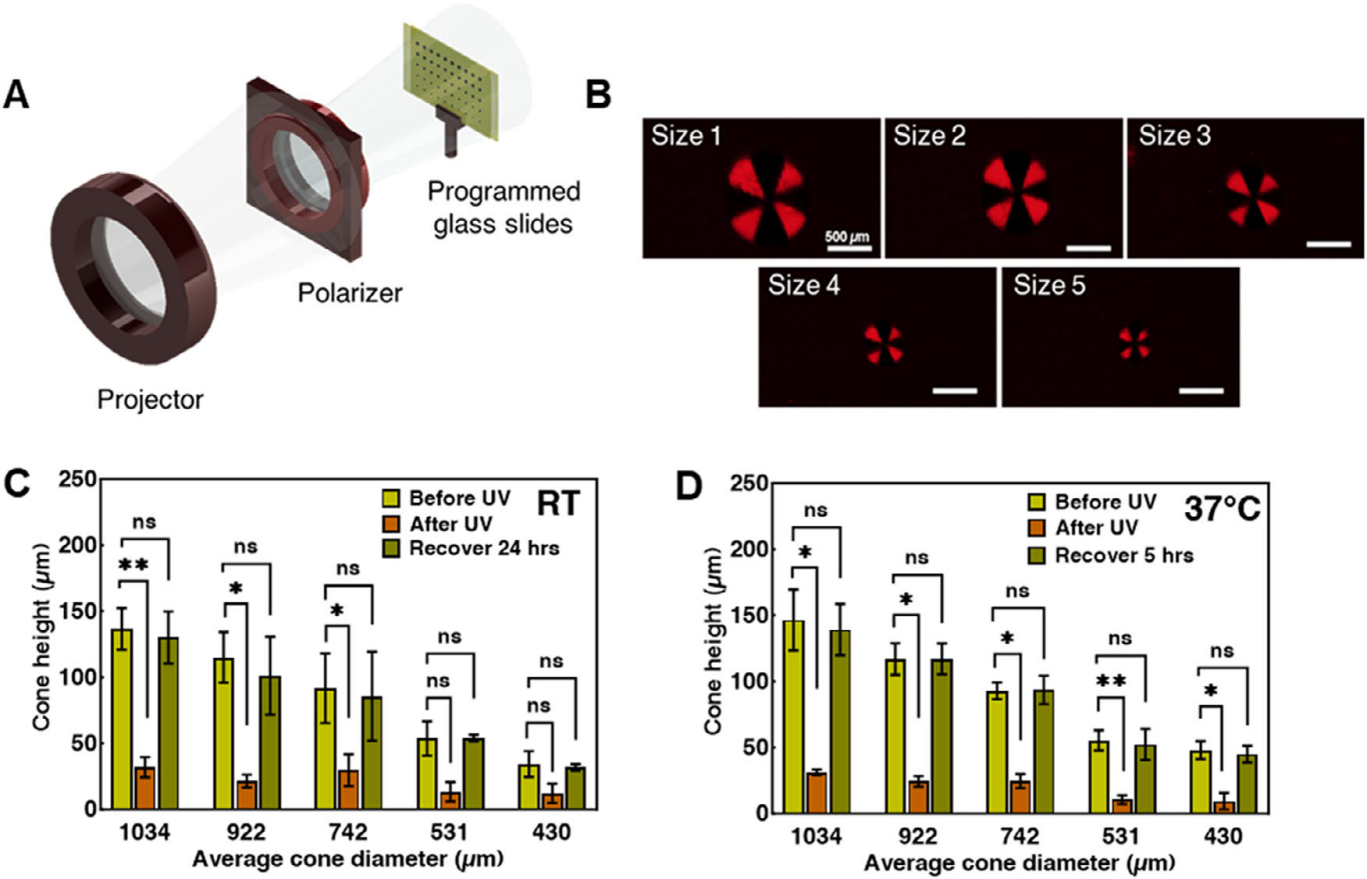
(A) Schematic of photoalignment setup (B) Polarized optical microscope images of varying diameters of patterns under cross polarizer (scale bar: 500 µm) (C) Cone height measurements before UV, after UV, and after recovering for 24 h at RT in an air environment. One‐way ANOVA was performed with Dunnett's comparison in each cone diameter group. (D) Cone height measurements before UV, after UV, and after recovering for 5 h at 37°C in air. One way‐ANOVA was performed with Dunnett's comparison within each cone diameter group. ns: *p* > 0.05, ^*^ *p* < 0.05, ^**^ *p* < 0.01. Data shown are representative of three replicates. Three cone samples are from individually synthesized batches. Data shown are the mean with error bars representing standard deviation (*n* = 3).

Deployment of sub‐mm structures can be achieved by spatially programming the nematic director in small domains. Five different diameters of a segmented approximation of radial +1 topological defect with eight discrete domains were programmed (Figure 4A,B) and made into LCNs. On cooling from the crosslinking temperature, the polymer films expand along the radius of the circle and contract along the azimuth, forming a cone [80]. The five patterns were varied from 1034 ± 26 µm (size 1), 922 ± 76 µm (size 2), 742 ± 19 µm (size 3), 531 ± 29 µm (size 4), and 430 ± 40 µm (size 5) (mean ± STD, *n* = 3). The actuated cone heights for each size are 139 ± 20 µm (size 1), 115 ± 11 µm (size 2), 78 ± 12 µm (size 3), 53 ± 11 µm (size 4), and 38 ± 10 µm (size 5) (mean ± STD, *n* = 3). The aspect ratios of each cone, as defined as the ratio of cone height to (base) diameter, were 0.134 (size 1), 0.125 (size 2), 0.105 (size 3), 0.100 (size 4), and 0.088 (size 5). This decrease in aspect ratio as a function of pattern size may indicate that the bending energy of the film becomes relevant to the deformation [81].

The cones formed by the LCNs can be nearly flattened by UV exposure and then recovered nearly fully in the dark at RT, across the range of cone sizes (Figure 4C). This pattern was present across all cone sizes, but the height of the smallest two cone sizes after UV exposure was not statistically different. All cones recovered fully, as indicated by a lack of statistical difference in height before UV exposure and after recovery. For a unique set of cones tested for recovery at physiological conditions, at 37°C in the dark, the actuated cones of each size recovered back to their initial cone height after 5 h. All cones after UV exposure showed a statistically significant decrease in height as compared to before UV exposure. All cones after recovering for 5 h showed no significant differences in height compared with the condition before UV exposure (*p* > 0.05) (Figure 4D), again indicating full recovery of the material.

### 2.4 Insertion and Buckling Force in Soft Tissues

**FIGURE 5:**
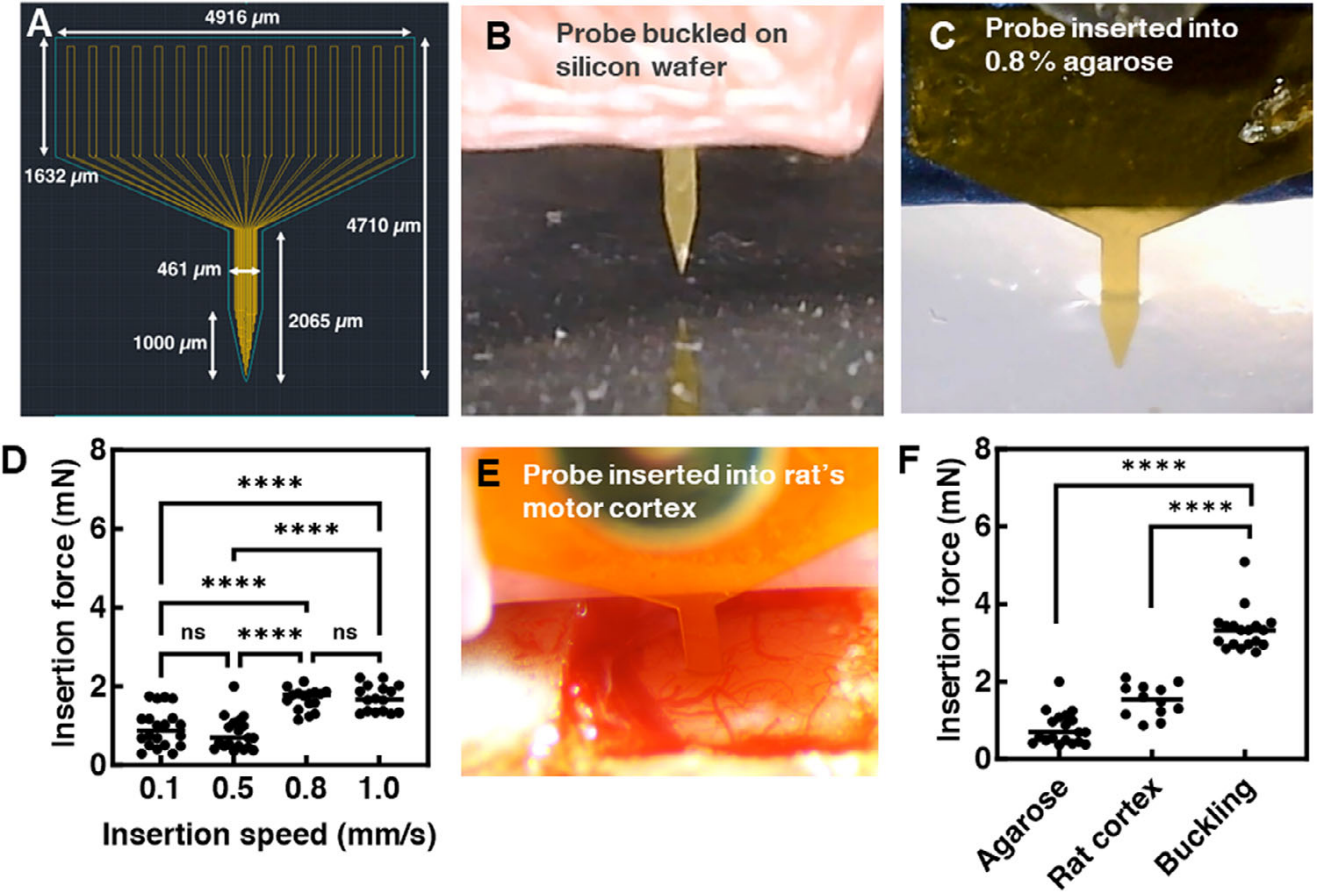
(A) Probe design, (B) image of in vitro critical buckling force tests on silicon wafer, (C) image of in vitro insertion force test into 0.8% agarose phantom, and (D) insertion forces tested in 0.8% agarose phantom at different insertion speeds, including 0.1, 0.5, 0.8, and 1.0 mm/s. One way‐ANOVA was performed with Tukey's comparison between each insertion speed. ns: *p* > 0.05, ^****^ *p* < 0.0001. (E) Image of in vivo insertion test into the rat motor cortex. (F) Insertion forces in agarose (at 0.5 mm/s) (the dataset is the same with 0.5 mm/s in (D)), rat motor cortex (at 0.5 mm/s), compared with in vitro buckling force results on silicon (at 0.01 mm/s). One‐way ANOVA was performed with Dunnett's comparison to buckling force. ^****^ *p* < 0.0001. Data shown are representative of 20, 19, 16, 16, 18, and 12 insertions for the conditions 0.1 mm/s, 0.5, 0.8, and 1.0 mm/s in agarose, 0.01 mm/s in silicon, and 0.5 mm/s in rat cortex, respectively. The data shown are the mean with error bars representing standard deviation for each condition.

ST/AZO/RM82 LCN‐based test devices may be suitable for surgical insertion into soft tissues, such as the brain, without the need for insertion tools. The ST/AZO/RM82 LCN devices were cut into dimensions similar in size to single‐shank intracortical microelectrode arrays [82, 83, 84] (Figure 5A). The probes were brought into contact with a stiff silicon wafer to measure the minimum force required to induce probe buckling (Figure 5B), which is 3.3 ± 0.5 mN (mean ± STD, *n* = 18). The probes were then inserted into a 0.8% agarose phantom, mimicking the brain tissue stiffness [85, 86, 87] (Figure 5C). The insertion forces in agarose phantom are 0.9 ± 0.5 mN (*n* = 20), 0.8 ± 0.4 mN (*n* = 19), 1.7 ± 0.3 mN (*n* = 16), and 1.7 ± 0.3 mN (*n* = 16) (mean ± STD) at different insertion speeds of 0.1, 0.5, 0.8, and 1.0 mm/s, respectively (Figure 5D). The insertion speed of 0.5 mm/s was chosen for performing in vivo insertion test. The devices readily inserted into rat motor cortex in vivo without buckling, at 0.5 mm/s, and the insertion force is 1.5 ± 0.4 mN (mean ± STD, *n* = 12) (Figure 5E). The force required for probe insertion, in both agarose (*p* < 0.0001) and rat motor cortex (*p* < 0.0001), was markedly less than the buckling force (Figure 5F), suggesting that the devices are robust to withstand the implantation process.

### 2.5 Deployment in Agarose Phantom

**FIGURE 6:**
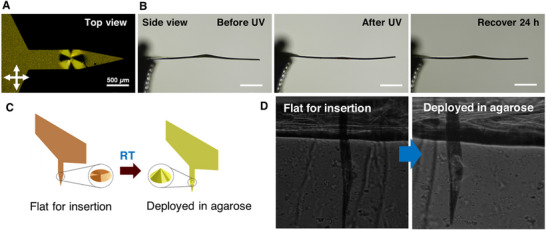
(A) Image of a probe with a patterned director between cross‐polarizers (top view). (B) Deployment of the probe in the air before and after UV exposure and recovery after 24 h at RT (side view). (Scale bar: 500 µm). (C) Schematics of the deployable probe being flattened for insertion and deployed in agarose after 24 h at RT. (D) Images showing deployable probes can be flattened for insertion and deployed back after 24 h at RT in 0.8% agarose (The width of the probe is 461 µm).

ST/AZO/RM82 LCN‐based devices deploy in the brain‐like agarose phantom. The director pattern needed to form a cone with an average diameter of 531 µm was programmed on the LCN films (Figure 6A). The molecular alignment outside the conical regions is controlled to be polydomain (Figure 6A). Polydomain alignment limits shape morphing within the remainder of the device. The micromachined probes demonstrated cone deployment at RT, can be flattened upon UV exposure, and recover back to cones in air (Figure 6B) as shown previously for films. When tested in agarose models, after UV exposure, the flattened probes can be inserted into agarose. After 24 h at RT in a brain‐like agarose phantom, the cone deployed to an average height of 61 ± 7 µm (mean ± STD, *n* = 3) (Figure 6C,D). This result supports the notion that the probes can not only be inserted into the brain‐like agarose phantom but also possess sufficient force to deploy within the agarose environment. The deployment distance is comparable to that associated with thicknesses of a foreign body response region (typically 50–100 µm) [12, 26, 34, 88] observed for in vivo implantation of neural probes. This implies that future devices, which employ this fabrication strategy, may be useful in deployable neural interfaces that could move electrodes away from the region of the most aggressive foreign body response. This work does not assess biocompatibility or evaluate material degradation in vivo. In vivo biocompatibility and material degradation studies are needed to evaluate the potential for the use of deployable LCNs in medical devices, such as neural interfaces.

## 3 Conclusion

In this study, we demonstrated the use of photoresponsive LCNs for potential use in biomedical devices that deploy hours after insertion without any need for a stimulus, including temperature change or water uptake. The type of shape change of the material can be programmed with directed self‐assembly of nematic monomers to patterned surfaces. With the incorporation of azobenzene, the photo‐responsive materials undergo *trans‐cis* isomerization upon UV exposure before implantation, which allows the device to be packaged into a metastable flat form. The shape change of the LCNs occurs spontaneously over 24 h at RT and 5 h at 37°C. Our results suggest that this approach may be suitable for deploying sub‐mm responsive structures over 50 µm, which is similar to the dimensions of FBR region. The most intense FBR region generally extends 50–100 µm from the device [12, 26, 34, 88]. Additionally, the LCN based test structures with dimensions based on conventional neural probes possessed adequate stiffness to penetrate soft tissues without buckling. Overall, we offer a new approach for the fabrication of self‐deploying devices for potential medical applications. Such an approach may enable future neural interfaces that deploy small structures within the brain.

## 4 Materials and Methods

### 4.1 Materials

The liquid crystal monomer mixture was prepared from monomers 6‐{4‐[4‐(Acryloyloxy‐hexyloxy)cyclohexyl]phenoxyhexyl acrylate (ST), 1,4‐Bis[4‐(6‐acryloyloxyhexyloxy)benzoyloxy]‐2‐methylbenzene (RM82), and 4,4'‐Bis(9‐(acryloyloxy)nonyloxy)azobenzene (AZO) from Synthon Chemicals GmbH & Co. KG. The liquid crystal monomer 1,4‐Bis[4‐(3‐acryloyloxypropyloxy)benzoyloxy]‐2‐methylbenzene (RM257) was purchased from Wilshire Technologies. A chain extender, n‐butylamine (nBA), was purchased from Tokyo Chemical Industry (TCI). Photo‐initiator phenylbis(2,4,6‐trimethylbenzoyl)phosphine oxide (I‐819) was purchased from TCI. Photo‐initiator 2‐Benzyl‐2‐(dimethylamino)‐4'‐morpholinobutyrophenone (I‐369) was purchased from TCI. Photo‐initiator 2,2‐Dimethoxy‐2‐phenylacetophenone (I‐651) was obtained from ACROS Organics. Brilliant yellow with a dye content of 85.2% was obtained from Chem‐Impex International. Solvents, dimethylformamide (DMF) and isopropanol, were purchased from Fisher‐Scientific. Acetone was purchased from Epredia through VWR International, LLC. Toluene was purchased from EMD Millipore (Merck). Agarose was purchased from Sigma‐Aldrich.

### 4.2 Mold Preparation and Photoalignment

Glass slides (50.8 mm × 76.2 mm × 1 mm) were cleaned with Alconox solution, acetone, and isopropanol and air‐dried, respectively. The glass slides were treated with plasma (Harrick Plasma, plasma cleaner model PDC‐001) and then baked on the 65°C hot plate until used. A 1 wt.% solution of brilliant yellow dye in DMF, was prepared and filtered by a 0.22 µm PTFE filter and deposited on the clean glass slides. Subsequently, the treated glass slides were spin‐coated at a speed of 3000 rpm and acceleration of 1000 rpm/s for 30 s. After spin coating, the glass slides were baked on the 65°C hot plate for at least 10 min.

For preparing glass slides for polydomain samples, the glass slides were spin‐coated with an LCN coating containing 10 wt.% of RM257 and 0.5 wt.% of photo‐initiator I‐651 in Toluene (LCN coating). The spin coating was performed at a speed of 1000 rpm and an acceleration of 450 rpm/s for 20 s. The coating was polymerized in a UV oven (UVP Crosslinker CL‐3000, Analytik Jena) with a UV intensity of 2 mW/cm^2^ at 365 nm for 30 min. After curing, two glass slides were assembled into a cell by placing a spacer (15 µm) between the glass slides and sealed with super glue.

For preparing glass slides for twisted nematic samples, a glass slide was placed on a setup with a projector (Vivitek, D912HD), a rotatable polarizer, and a slide holder as previously described [89]. Linearly polarized light with an intensity of 2.3 mW/cm^2^ was utilized to photo‐align the dye on one glass slide along the long‐axis for 15 min, and another glass slide was photo‐aligned along the short‐axis for 15 min. These two slides would be used to assemble a cell. After photoalignment, both glass slides were spin‐coated with LCN coating and cured in a UV oven as described above.

For preparing glass slides for cone samples, the slides were overlapped and placed on the same setup as described. The slides are then photo‐aligned with a director pattern associated with a radial +1 topological defect with eight discrete domains as described in a previous study [60, 80]. Each domain was photo‐aligned for 20 min. After photoalignment, both glass slides were spin‐coated with LCN coating and cured as described above. After curing, the glass slides were assembled under a polarized optical microscope (Nikon, Eclipse LV100N POL) to ensure the pattern on each slide was aligned.

### 4.3 LCN Synthesis

Three different LCN samples were considered in the present study, identified by the diacrylates used for synthesis, as RM82, AZO/RM82, and ST/AZO/RM82 LCNs. The molar ratio of each component across samples was summarized in Table 1 with 0.8% of photoinitiator I‐369 or I‐819. For RM82 LCNs, monomer RM82, photoinitiator I‐369, and nBA were heated until the RM82 melted, mixed using a vortex mixer, and filled into the prepared cells. The sample was oligomerized overnight at 80°C, undergoing the aza‐Michael addition. Then, the sample was photocrosslinked at 80°C (25 mW/cm^2^, UV light at 365 nm) (OmniCure, LX500). One side of the sample was exposed to UV for 20 s and flipped to the other side for another 20 s under UV light exposure. The same procedure was then repeated for the following times: 20 s, 20 s, 30 s, 1 min, 3 min, 5 min, 10 min, 30 min, and 45 min in sequence. For AZO/RM82 LCNs, monomers AZO and RM82, photoinitiator I‐819, and nBA were heated and filled into the prepared cells as described above. The sample was oligomerized overnight on the 80°C hotplate, undergoing the aza‐Michael addition. Then, the sample was photocrosslinked at 80°C (90 mW/cm^2^, UV light at 400 nm) (OmniCure, LX500). One side of the sample was exposed to UV for 20 s and flipped to the other side for another 20 s under UV light exposure. Next, the same procedure was then repeated for the following times: 20 s, 20 s, 30 s, 1 min, 3 min, 5 min, 10 min, 30 min, 45 min, and 60 min, sequentially. For ST/AZO/RM82 LCNs, monomers ST, AZO, and RM82, photoinitiator I‐819, and nBA were heat‐melted together and filled into the prepared cells. The sample was oligomerized overnight on the 145°C hotplate, undergoing the aza‐Michael addition. Then, the sample was free‐radically crosslinked at 160°C (90 mW/cm^2^, UV light at 400 nm) (OmniCure, LX500). The sample was crosslinked the same way as AZO/RM82 LCNs. After cross‐linking the sample, the cells were gently opened by a blade.

**TABLE 1:** The molar ratio of each composition.

Sample name	Molar ratio				
	Acrylate/amine	ST	RM82	AZO	nBA
RM82	2.3	x	2.30	x	1
AZO/RM82	2.3	x	1.92	0.38	1
ST/AZO/RM82	2.3	1.30	0.62	0.38	1

### 4.4 Gel Fraction

Gel fraction was performed using our previously established procedures to confirm samples are well crosslinked [60]. Each sample was weighed for its initial mass (*M_initial_*) and then placed in 20 mL of chloroform for 48 h. The sample was air‐dried for 24 h at RT and then placed in a vacuum oven for 48 h at 60°C. The sample was set in the air for 2 h before taking the weight. Next, each sample mass was weighed to determine the final mass (*M*_*final*_) (n = 3). The final gel fraction percentage was calculated using the following equation:

$$Gel\;Fraction\;\left(\%\right)=\frac{M_{final}}{M_{initial\;}}\times 100\%$$

### 4.5 Mechanical Characterization

Dynamic mechanical analyzer (TA Instruments, RSA‐G2) was used to measure storage modulus and tan delta in tension as a function of temperature. The minimum axial force was set at 10^−4^ N. The axial force was otherwise applied 75% higher than the dynamic force. The sample was heated from −30°C to 150°C at a rate of 3°C/min. The applied strain on the sample is 0.2% at a frequency of 1 Hz. Polydomain samples were cut into dimensions of 26.8 mm in length and 4.8 mm in width. The test was performed three times for each composition (*n* = 3). The glass transition temperature (*T*_g_) was denoted as the peak of tan delta.

### 4.6 Thermal Characterization

The nematic‐to‐isotropic temperature (*T*_NI_) of monomer solutions and oligomers was tested using polarized optical microscopy (POM) (Nikon, Eclipse LV100N POL). A red filter was placed above the light source on the POM to prevent photopolymerization. Samples were observed between crossed polarizers. The monomer solution was applied on a glass slide covered by a coverslip and then heated to its isotropic phase on a thermal stage (Linkam, LTS420) at a heating rate of 30°C/min. Once reaching the isotropic phase, the monomer mixture was cooled at a cooling rate of 5°C/min. The onset of birefringence on cooling POM was recorded as *T*_NI_. *T*_NI_ are reported in mean ± standard deviation with 3 replicates.

### 4.7 Curvature Measurement of Benders in Air and Saline

We measured curvature to evaluate the photo‐responsiveness and recovery time of the twisted nematic ST/AZO/RM82 samples. Twisted nematic LCN samples were cut into dimensions of 3 mm in length and 1 mm in width. The cut sample was then heated over *T*_g_, to 80°C to remove the thermal history, and then cooled to RT. The sample was placed under UV light (365 nm) with an intensity of 115 mW/cm^2^ in air. The sample was first exposed to the UV light for 1 min and then flipped to the other side for another 1 min of UV exposure. The procedure was repeated 3 times for each sample. The shape of the sample was then photographed over time at RT and 37°C in air and at 37°C in saline (VWR Life Science, phosphate‐buffered saline). Pictures were captured at the time points: before exposing UV, after exposing UV (T_0_), recovery for 5 min, 10 min, 30 min, 1 h, 2 h, 3 h, and 5 h at 37°C, and collected at the same time points but with one more time point at 24 h for recovering at RT. The images were taken when blue and green lights were filtered. After taking the images, the samples were stored in the dark. Image J was used for data analysis to fit a circle to the bent LCNs samples, where:

$$Curvature\;\left(1/mm\right)=\frac{1}{radius\;of\;the\;fitted\;circle}$$

The curvatures are reported in mean ± standard deviation with 3 replicates for each condition (*n* = 3).

### 4.8 Water Uptake

A water uptake study of LCN films was performed in saline (VWR Life Science, phosphate‐buffered saline) at 37°C. Three ST/AZO/RM82 LCN films were cut into the dimensions of 3.7 cm × 2.8 cm. Each sample was soaked in 100 mL of saline. Before taking wet mass measurements, the LCNs were blotted dry twice using a paper towel for 30 s. The wet mass was recorded for 7 days. After the 7‐day study, the samples were dried in 37°C oven for 6 days, and the dry mass was measured. The wet mass and dry mass changes are reported in mean ± standard deviation with 3 replicates for each condition (n = 3).

### 4.9 Actuation Measurement of Cones in Air

Five sizes of the +1 defect patterns were programmed to study the cone actuation and recovery time at RT and 37°C. The monomer mixture of ST/AZO/RM82 was used. The cone sample was cut and heated over *T*_g_ (50°C) to remove the thermal history, allowing the cone actuation. Then, the sample was taped on the edges to a glass. A ‘Z‐stack’ optical measuring feature on POM was used to measure the cone height. A red filter was used for POM setup to avoid possible recovery by exposing it to white light. The cone sample was exposed under the UV light (115 mW/cm^2^, 365 nm) for 5 min. The cone height was measured during recovery at both RT and 37°C in air. At RT, the cone height was assessed before UV exposure, after UV exposure, and after recovering at RT for 24 h. At 37°C, the cone height was tested before UV exposure, after UV exposure, and after recovery at 37°C for 5 h. During the measurement, the blue and green lights were filtered. All samples were covered with foil when they were not under analysis. There were 3 replicates for these tests. The cone heights are reported in mean ± STD with 3 replicates for each condition.

### 4.10 Laser Cutting

A laser cutter (LPKF ProtoLaser, U4) was used to machine the LCN probe geometries from the LCN films while on the glass substrate. An outline of the probe layout was created on the LCN substrates for insertion tests. Following laser cutting, a drop of tap water was used to release the probe from the glass slides. The overall dimensions of the probe on the AutoCAD file were 4916 µm in length and 4710 µm in width. The dimensions of the shank were 2065 µm in length and 461 µm in width. To obtain deployable probes, +1 director patterns with an average diameter of 531 µm were first programmed on the LCN films.

### 4.11 Insertion Force and Buckling Force Testing

An in vitro insertion force test was performed with a 0.8% agarose gel. The ST/AZO/RM82 LCN probes were connected to a force transducer (Miniature S‐Beam Jr. Load Cell, FUTEK). The agarose gel was placed beneath the inserter, transducer, and probe. Probes were inserted into the gel at 0.1, 0.5, 0.8, and 1.0 mm/s to a depth of approximately 1.5 mm. These speeds were selected based on previous studies [90, 91, 92, 93, 94, 95].

In addition, an in vitro buckling force test was performed and repeated using the above procedure but with agarose gel replacing the silicone‐coated silicon wafer. To determine the buckling force in air, the probe was compressed against the wafer at a speed of 0.01 mm/s, forcing the probe to buckle. The slow speed (0.01 mm/s) was chosen to ensure that it was possible to observe the time and record the buckling force accurately. The transducer recorded the force continuously, and the entire session was captured on video. The force trace and the recorded videos were used to identify the time of contact with the wafer and the time of buckling and associated buckling force.

For in vivo tests, all procedures were approved by The University of Texas at Dallas Institutional Animal Care and Use Committee (Approval No. 20‐06). Surgery was performed using previously described procedures [96]. Adult Sprague Dawley rats were used, including one male and one female. Anesthesia was induced and maintained using 2%–3% isoflurane combined with medical‐grade oxygen. The animals were first placed in a stereotactic frame. Then, once anesthetized, a midline incision was made on the scalp, followed by tissue resection to expose the skull. Afterward, a ∼3 mm × 3 mm craniotomy and subsequent durotomy were performed over the left motor cortex. Then, the transducer was connected to the same implant inserter. Probes were inserted into the cortex at 0.5 mm/s. Each probe was inserted into the brain three times at different locations, targeting areas lacking large surface‐level blood vessels (when possible) to minimize variability between insertions. Three total insertions were performed in the male animal, and nine total insertions were performed in the female animal, using a total of four probes. The procedure was captured on video, and the transducer recorded force continuously. Synchronization of the video and force recordings was used to identify the time of penetration of the cortex.

### 4.12 Deployment Measurement of Cones in Agarose

The +1 defect patterns with an average cone diameter of 531 µm were programmed on the ST/AZO/RM82 LCN films. The patterns were placed in the desired position of the shank. The films were then cut into a desired probe dimension by laser (LPKF ProtoLaser, U4) with the same parameters as mentioned. Tap water was applied to the surface of the sample to easily detach the probe from the glass slides. After removing the thermal history, the detached probes were able to show a cone‐shaped deployment on the shank. The deployable probes were flattened by UV exposure (115 mW/cm^2^, 365 nm) for 5 min and then inserted into 0.8% agarose. After 24 h in agarose, the deployable probes were deployed in their original cone shape. The cone height in agarose was captured from the side view by a microscope. The cone heights in agarose are reported in mean ± STD with 3 replicates.

### 4.13 Statistics

All statistics were performed using GraphPad Prism (version 10.1.2). For comparing three different test conditions, one‐way ANOVA was used with either Tukey's or Dunnett's comparison. Details are provided in the corresponding caption. For comparing two different test conditions, a two‐tailed unpaired t‐test was conducted assuming the datasets follow a normal distribution. For comparing water uptake, a paired t‐test was used. *p* < 0.05 was regarded as a statistically significant difference. p > 0.05 was viewed as a non‐significant difference. *p < 0.05, **p < 0.01, ***p < 0.001, and ****p < 0.0001.

## Funding

National Institute of Neurological Disorders and Stroke of the National Institutes of Health under Project No. 1R01NS131502 (T.H.W., J.J.P., and J.R.C.). Department of Defense Office of Naval Research Award No. N00014‐23‐1‐2225 (Hatice Ceylan Koydemir).

## Conflicts of Interest

The authors declare no conflicts of interest.

## Data Availability

The data that support the findings of this study are available from the corresponding author upon reasonable request.
